# Anonymize or synthesize? Privacy-preserving methods for heart failure score analytics

**DOI:** 10.1093/ehjdh/ztae083

**Published:** 2024-11-20

**Authors:** Tim I Johann, Karen Otte, Fabian Prasser, Christoph Dieterich

**Affiliations:** Klaus Tschira Institute for Integrative Computational Cardiology, University Hospital Heidelberg, Im Neuenheimer Feld 669, 69120 Heidelberg, Germany; Berlin Institute of Health, Charité, Universitätsmedizin Berlin, Charitéplatz 1, 10115 Berlin, Germany; Berlin Institute of Health, Charité, Universitätsmedizin Berlin, Charitéplatz 1, 10115 Berlin, Germany; Klaus Tschira Institute for Integrative Computational Cardiology, University Hospital Heidelberg, Im Neuenheimer Feld 669, 69120 Heidelberg, Germany; German Center for Cardiovascular Research (DZHK), Internal Medicine III, Partner Site Heidelberg/Mannheim, Im Neuenheimer Feld 669, 69120 Heidelberg, Germany

**Keywords:** Cardiology, Data sharing, Data privacy, Risk scores

## Abstract

**Aims:**

Data availability remains a critical challenge in modern, data-driven medical research. Due to the sensitive nature of patient health records, they are rightfully subject to stringent privacy protection measures. One way to overcome these restrictions is to preserve patient privacy by using anonymization and synthetization strategies. In this work, we investigate the effectiveness of these methods for protecting patient privacy using real-world cardiology health records.

**Methods and results:**

We implemented anonymization and synthetization techniques for a structure data set, which was collected during the HiGHmed Use Case Cardiology study. We employed the data anonymization tool ARX and the data synthetization framework ASyH individually and in combination. We evaluated the utility and shortcomings of the different approaches by statistical analyses and privacy risk assessments. Data utility was assessed by computing two heart failure risk scores on the protected data sets. We observed only minimal deviations to scores from the original data set. Additionally, we performed a re-identification risk analysis and found only minor residual risks for common types of privacy threats.

**Conclusion:**

We could demonstrate that anonymization and synthetization methods protect privacy while retaining data utility for heart failure risk assessment. Both approaches and a combination thereof introduce only minimal deviations from the original data set over all features. While data synthesis techniques produce any number of new records, data anonymization techniques offer more formal privacy guarantees. Consequently, data synthesis on anonymized data further enhances privacy protection with little impacting data utility. We share all generated data sets with the scientific community through a use and access agreement.

## Graphical Abstract

**Graphical Abstract:**
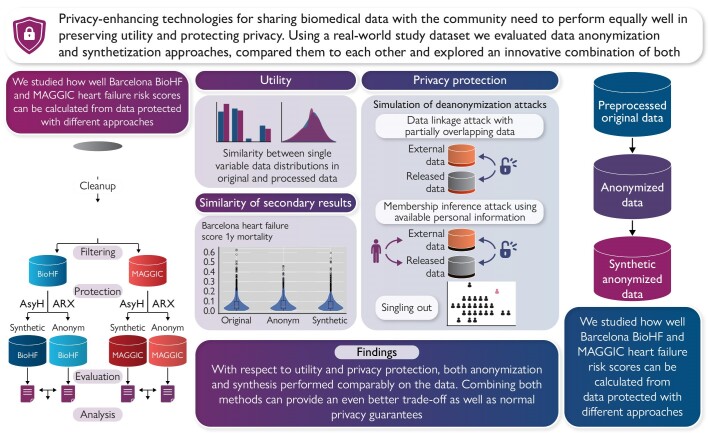

## Introduction

The crucial role of data science and machine learning (ML) for the future of medicine has been widely recognized.^1,2^ To create the large data sets needed for data-driven discoveries, data need to be re-used beyond its initial scope of collection and shared across institutional and national boundaries.^3^ In cardiology, predicting risk of re-hospitalization or death in patients with heart failure (HF) is crucial for optimizing individualized preventive, diagnostic, and therapeutic strategies, as well as for the efficient allocation of healthcare resources. Sharing data, which was used to develop or validate risk scores, is vital to drive continuous improvements in risk modelling. However, medical data are privacy protected and cannot be freely shared. Insufficient privacy may have severe consequences, such as identity theft, financial fraud, and compromised patient care.^4^ Consequently, there is a growing emphasis on the development of technologies for securely sharing clinical data.^5^ Many different approaches have been suggested, ranging from federated learning where data remain at the participating institutions^6^ to methods for generating privacy-preserving versions of a given data set. The latter is the focus of this paper.

Generating protected data sets is complex, as simply removing directly identifying properties, such as names or insurance numbers, is usually not enough to prevent malicious recipients from linking data to other data sets or deriving sensitive properties of individuals.^7^ Brute force approaches such as ‘blurring’ or randomizing data can impact its statistical properties and hence reduce its utility. That is why methods for creating protected data sets are generally subject to an inherent risk/utility trade-off.^8^ Traditional methods for protecting clinical data encompass anonymization techniques, such as generalization, suppression, or randomization of values that could be used to re-identify patients, often combined with risk models such as k-anonymity to create groups of indistinguishable data records.^9^ More recently, ML methods emerged to generate synthetic data that preserve the underlying statistical properties of the training data provided.^10^ While there is evidence that synthetization can provide a better trade-off between privacy protection and output data utility,^11,12^ especially in scenarios with high-dimensional data, synthetic data do not always capture all relevant statistical properties and are not free from residual privacy risks. Some researchers have argued that synthetization provides few benefits over traditional anonymization methods.^13^ Evidently, both approaches could be combined by using anonymized data to train synthetization models or vice versa.

Measuring the utility of a protected data set is challenging in itself, and a wide range of general-purpose metrics have been proposed.^14^ If potential applications of a protected data set are known in advance, its utility can be measured by how well the derived results obtained with unprotected data agree with those derived from protected data.^14^ This is particularly important for ML tasks, as training models with protected data can also ensure that the models themselves are privacy preserving.^15^

The quantitative assessment of privacy risks in anonymized data sets is well established.^16^ However, the evaluation of synthetic data has only recently gained attention, following studies, which demonstrated the potential to extract information about individuals, whose data were used to train the synthetization models.^13,17^ Few frameworks for quantitative risk assessment allow the evaluation of anonymized as well as synthetic data that make a comprehensive assessment and comparison of risks challenging.^18^

We aim to explore the effectiveness of anonymization and synthetization methods for sharing cardiology data collected from clinical routine care. More specifically, we apply anonymization and synthetization methods to clinical data, evaluate residual privacy risks, and study the utility of the protected data sets by computing two HF risk scores. Our contributions are three-fold: (i) We developed specific implementations of anonymization and synthetization methods for cardiology data as well as an innovative combined approach, (ii) we performed a comparison of the risk-utility trade-off achieved by these methods providing insights into potential benefits and shortcomings of synthetization and anonymization, and (iii) we created data sets where privacy is protected by one or two layers of the aforementioned methods and openly share them with the scientific community through a data use and access agreement.

Our software and open data sets fulfil all Findable, Accessible, Interoperable, Reproducible (FAIR) criteria as outlined under https://www.go-fair.org/fair-principles/.

## Methods

**Figure 1:**
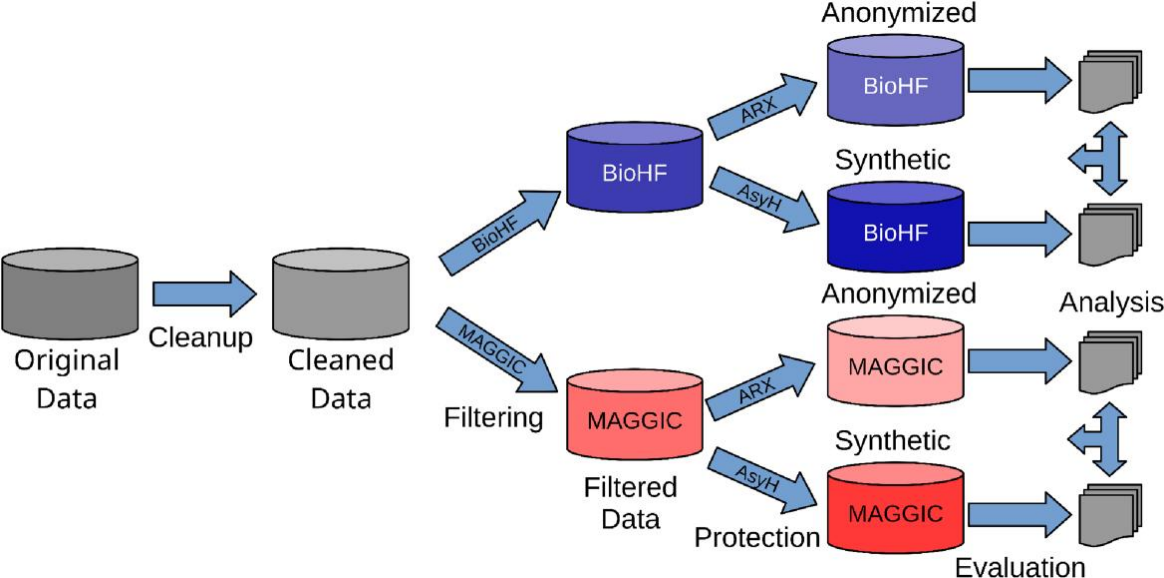
The anonymize and synthesize workflow implemented for data preparation, protection, and analysis (https://github.com/dieterich-lab/AnonymizeAndSynthesize). From the pre-processed cleaned data Meta-Analysis Global Group in Chronic Heart Failure and Barcelona Bio Heart Failure scores are calculated. The records with calculable score values are separated to their respective filtered data sets, from which (i) an anonymized data set is created using the ARX Data Anonymization Tool and (ii) a synthetic data set is generated using the Anonymizing Synthesizer For Health Data. ASyH, Anonymizing Synthesizer For Health Data; BioHF, Bio Heart Failure; MAGGIC, Meta-Analysis Global Group in Chronic Heart Failure.

We implemented an open-source software workflow, which executes all steps including pre-processing, anonymization, synthesis, statistical analysis, and final evaluation of the protected data sets (cf. *Figure 1*). In the following, we describe the respective methods step by step.

### Study data

Our data originate from a multi-centre cardiology study^19^ and consist of 2441 records with 18 variables containing personal data and patient history (*n* = 9), medication (*n* = 4), and lab results (*n* = 5; see *Table 1*). This data set was previously used to calculate the Barcelona Bio Heart Failure (BioHF)^20^ and the Meta-Analysis Global Group in Chronic Heart Failure (MAGGIC)^21^ risk scores, which predict survival of *HF* patients after hospitalization. Some of the variables are shared between the two formulae to compute the scores, such as the patients’ age and gender, their medication with beta-blockers and angiotensin-converting enzyme inhibitor and aldosterone receptor blocker, their New York Heart Association (NYHA) dysfunctional class, and left ventricular (LV) ejection fraction. Some of the variables that are necessary to calculate these risk scores are known to be associated with a risk of re-identification, such as those in demographics and patient history,^7^ which renders the data set subject to privacy concerns, presenting an obstacle to its dissemination beyond the initial scope of collection.

**Table 1:** Data categories and individual variables in the study data set [19]

Category	Variable
Demographics	Age (B, M) Sex (B, M)
Patient history	BMI (M) Systolic blood pressure (M) NYHA dysfunctional class (B, M) Current smoking (M) Time of first diagnosis of HF (M) Diabetes mellitus (M) Chronic lung disease (M)
Medication	Beta blocker (B, M) ACEi/ARB (B, M) Statin (B) Loop diuretic (B)
Echocardiography	LV ejection fraction (B, M)
Laboratory data	Creatinine (M) Sodium (B) Haemoglobin (B) Estimated GFR (B)

‘B’ and ‘M’ indicate whether a variable is needed for calculating the Barcelona BioHF for MAGGIC heart failure score, respectively.

ACEi, angiotensin-converting enzyme inhibitor; ARB, aldosterone receptor blocker; BMI, body mass index; GFR, glomerular filtration rate; LV, left ventricular; NYHA, New York Heart Association.

### Data cleaning and pre-processing

The original data have been cleaned up by first removing outliers, i.e. replacing values outside a plausible range for the respective variable with N/A indicators. The ranges correspond to the ones used in the HiGHmed study.^19^ As a consequence, fewer records could be used for this study than in the original work,^19^ as scores were only computed for complete data records.

The pre-processed data were divided into two subsets, one contained all records with calculable BioHF scores, and the other consisted of all records with calculable MAGGIC scores, which resulted in tables of 1326 records with 11 variables and 890 records with 13 variables, respectively. Both risk scores were calculated with the same R scripts as in Sommer *et al*.^19^

### Measuring the utility of protected data

We estimated data utility by comparing univariate distributions of variables as well as risk score distributions for all derived data sets with the respective distributions of the original data. The ability to reproduce those score distributions is a suitable proxy for data utility as underlying variable dependencies need to be preserved. The comparison was performed using visualizations, including violin plots and plots of the estimated cumulative distributions, and statistical tests, including two-sample Kolmogorov–Smirnov test for continuous variables, *χ*^2^ tests for categorical variables as well as Cohen's *d* for effect sizes. Non-uniform entropy was employed to assess similarity between variables that have been protected through generalization.

### Measuring and protecting privacy

When assessing privacy risks, it is common to distinguish between *identity disclosure*, often also called re-identification, which is the process of successfully associating a data record with the originating individual, *attribute disclosure*, which is the process of revealing inherent, individual characteristics, even without direct re-identification, and *membership disclosure*, which is the process of revealing the presence of an individual in the protected data set.^22^ In the context of a cardiology data set from routine care, a re-identification would, for example, be the identification of an individual person that the attacker personally knows or a prominent personality treated in the hospital. Attribute disclosure would reveal sensitive information like the disease severity state from demographic properties, whereas membership disclosure would reveal that an individual was included in the data set.

Typically, data anonymization and synthetization only explicitly protect from identity disclosure. In case of anonymization, this is due to the conceptual challenges around the protection from other types of disclosure and the potential severe impact on data utility.^23^ Data synthesization, in turn, protects against identity disclosure by design, as it generates artificial records that usually cannot be linked back to specific individuals. However, attribute and membership inference can also be performed using synthetic data.^13,17^ Specifying protection guarantees from attribute and membership disclosure *a priori*, e.g. by integrating differential privacy into the training process for synthetization models^24^ or t-closeness and δ-presence into anonymization processes,^25,26^ usually leads to overprotection and impacts output data utility too much.^23^ Hence, a common approach, which we also pursue in this work, is to focus on identity disclosure as an upfront guarantee and to evaluate the residual risk of attribute and membership inference *a posteriori*, i.e. after the data protection mechanism was applied.

In this work, we use two common approaches to measure risks: (i) the Anonymeter framework by Giomi *et al.*^27^ and (ii) the hold-out approach suggested by Platzer *et al.*^28^ Both approaches quantify risks by splitting the input data set into a *hold-out or control data set* and a *training data set*, which is then protected using anonymization or synthetization. We used a split of 1:1 between training and hold-out/control for both frameworks.

The Anonymeter framework randomly generates *attacks* performing singling out and linkage (which are both individual steps of identity disclosure) and inference (i.e. attribute disclosure). An attack is carried out by randomly choosing certain types of information (e.g. a combination of values for different attributes) from the protected data set and checking whether this information can also be found in the control data set or the training data set. As a result, the framework provides risk *scores* for the different types of attacks with values between 0.0 and 1.0, which describe the success rate of attacks on the training data set normalized by the success rate of the attacks on the control data set.^27^ We configured Anonymeter to perform 400 attacks per attack scenario. Moreover, as the risk assessment process is randomized, we repeated it 50 times to study whether the results are stable and report average risk scores with standard deviations.

The hold-out approach complements the residual risk assessment by studying the distance between the protected records and the records in the training or hold-out data set, which could, for example, be used to perform membership attacks. Privacy risks are estimated by dividing the average distance to the training data set with the average distance to the hold-out data set [called *median distance-to-closest-record (DCR) ratio*] and by determining the fraction of records from the protected data set, which are closer to the training than to the hold-out data set (called *share*). For the median DCR ratio, the privacy risk increases as the deviation from 1.0 becomes more pronounced. Similarly, for the share metric, the privacy risk increases with greater deviations from 0.5.

#### Data anonymization with ARX

Data anonymization is the process of altering records, variables, and values in a data set that could be used for direct or indirect identification of a person. This is usually done by specifying how privacy risks and output data utility should be quantified and then performing an optimization process that applies modifications like deletions, generalizations, or aggregations to the data set while aiming for an optimal trade-off.

Our data anonymization process was implemented in Java using the software library as provided by the ARX Data Anonymization Tool.^29^ For each type of HF risk score, the data were anonymized in such a way that for each record, all demographic and clinical parameters were indistinguishable from the values of at least one other record (i.e. *k* anonymity with k=2).^7^ As described above, this optimization goal focuses on reducing the risk of identity disclosure. The parameter *k* should always be greater than 1 and the lowest possible value of k=2 was chosen, because of the relatively low number of records in the data sets, which makes it hard to achieve indistinguishability, and because it only defines the *a priori* privacy guarantee, while a posteriori protection is evaluated separately.

During the anonymization process, numerical values were clustered, and values were replaced with the geometric mean within each cluster. For the NYHA score, generalization to lower severity (Score I or II) and higher severity (Score III and IV) was allowed when deemed necessary by the optimization. Binary parameters were protected by removal of individual entries (cell suppression), whereas all parameters that were not required for the score calculations were removed from the data set (attribute suppression). ARX’s local optimization algorithm was used with 1000 iterations and 50 000 steps of the best-effort, bottom-up search strategy^30^ and the goal of minimizing the degree of fuzziness introduced.^31^

#### Data synthetization with ASyH

Data synthetization refers to the process of creating artificial data, which closely resembles the original data from which it was derived. In this manuscript, we achieve this by training artificial intelligence models that generate variations of the training data mimicking the statistical properties observed in the training data set itself. The most widely used models consist of ML algorithms fitting statistical distribution, specifically generative adversarial networks^32^ and variational autoencoders.^33^

For producing our synthetic data set, we deployed an ASyH pipeline. ASyH^34^ is a utility for automatic generation of synthetic tabular data using ML and deep learning (DL) algorithms provided by the Python suite Synthetic Data Vault (SDV).^35^ The ASyH pipeline will choose the best-performing ML/DL model by first training each of the four SDV models (Gaussian copula model, tabular variational autoencoder, conditional tabular GAN, and Gaussian copula GAN^35^) on the original data set, generating test data sets from each, and evaluating them with regard to their statistical fidelity compared with the input data set.

This fidelity test consists of a cumulative score calculated by a set of metrics defined in the SDMetrics’ ‘Quality Report’: *column shapes* and *column pair trends*. *Column shapes* compares the data distribution within a column in the synthetic data set with the corresponding column in the original data set. For numerical and date/time data, this is done by calculating the Kolmogorov–Smirnov ‘complement’, i.e. 1−d where *d* is the Kolmogorov–Smirnov statistic. For columns with Boolean or categorical data, the complement of the *total variation distance* (*TVD*), 1−δ(R,S), is used, in which the TVD is simply defined by the sum $\delta (R,\;S)=\frac{1}{2}\sum_{\omega \in \text{Ω}}\;\left|R_{\omega }-S_{\omega }\right|$ over all possible categories *ω* of *Ω*, where $R_{\omega }$ is the frequency of category *ω* in the original data and $S_{\omega }$ the corresponding frequency in the synthetic data set. *Column pair trends* is a method to compare inter-column correlations. For each pair of columns in the original data set, a distribution similarity value is calculated and compared with the corresponding value within the synthetic data set. For pairs of two numerical or date/time columns, the correlation similarity is used, defined by the formula $S=1-\frac{1}{2}|\rho_{R}(A,\;B)-\rho_{S}(A,\;B)|$, in which $\rho_{Q}(A,\;B)$ is the Pearson correlation between pairs of Columns A and B within data set Q. For the more frequent case of mixed pairs of numerical/date and Boolean/categorical data types as well as pairs of Boolean/categorical only, the contingency similarity is used. It is defined by $T=1-\frac{1}{2}\sum_{\alpha \in A}\sum_{\beta \in B}\sigma_{S}(\alpha ,\;\beta )-\sigma_{R}(\alpha ,\;\beta )$, where A and B are the sets of possible categories (or bins in the presence of one numerical/date column) and $\sigma_{Q}(\alpha ,\;\beta )$ the frequency of pairs with values (α,β) within data set Q. The above fidelity scores are normalized and so the averaged value yields an unweighted normalized value used for the overall fidelity evaluation. The model whose test data set yielded the best fidelity score is saved and a new synthetic data set is generated from this model as the resulting synthetic data set.

#### Combining anonymization and synthetization

One drawback of the type of data synthetization algorithms used in this work, compared with data anonymization based on mathematical privacy models, is the lack of a formal protection guarantee. The guarantee employed in our anonymization method that individuals are indistinguishable from others is one common and easy to understand approach. At the same time, data sets anonymized with this approach will be quite homogeneous, consisting of groups of indistinguishable records, while the synthetization method generates more heterogeneous and nuanced records.

A combination of both approaches, which can be constructed by first anonymizing the data set and training a synthesization model with the anonymized data set, could overcome the limitations of both approaches. Subsequent utility and privacy risk analysis is performed analogously to the single-method cases.

## Results

For both risk score scenarios, we developed tailored anonymization and synthetization procedures as well as a combination thereof. Subsequently, we compared their utility and residual risks.

### Data utility

We used the two-sample Kolmogorov–Smirnov test to quantify similarity of the calculated risk scores. This was later also applied to individual continuous input variables and the *χ*^2^ test was used for categorical and Boolean variables (cf. Supplementary material online, *Section S2.1*).

We identified 1326 out of 2441 records in the original data set for which the BioHF could be computed and used this subset to perform anonymization and to train data synthesization models. The anonymization process reduced the data set to 1324 records (i.e. suppressing two records), while synthesization by design resulted in no reduction.

A detailed comparison of all input variables (see Supplementary material online, *Table S1*) for both risk scores regarding their preservation of statistical properties is presented in Supplementary material online, *Figure S1* and *Table S2* for the BioHF score and in Supplementary material online, *Figure S2* and *Table S3* for the MAGGIC score. In brief, neither anonymization nor synthetization nor their combination perfectly agrees for all six continuous variables. Sodium and haemoglobin levels are significantly different in both privacy-preserving strategies [sodium mean (standard deviation) original: 139.17 (3.25) mmol/L, synthetic: 139.14 (3.23) mmol/L, anon.: 139.16 (2.70) mmol/L, Kolmogorov–Smirnov test *P* ≤ 0.005, haemoglobin levels original: 13.07 (2.10) g/dL, synthetic: 12.98 (2.10) g/dL, anon.: 13.02 (1.79) g/dL, *P* ≤ 0.005]. Moreover, age and LV ejection fraction additionally differ significantly from the original data for the anonymization approach, mainly due to a reduction in standard deviation [age mean (standard deviation) original: 67.47 (14.28), anon.: 67.03 (13.42), *P* = 0.009, *d* = 0.032]. The combined protection data set shows significant differences in all variables except for *estimated glomerular filtration rate*.

Intriguingly, the effect sizes of all significant deviations as expressed by Cohen’s *d* value are small to very small for any privacy-preserving method (Cohen’s *d* < 0.1).

Next, we compared the distributions of the six relevant categorical variables for BioHF score computation with the original data. Herein, we noticed that the NYHA variable differed significantly for the synthetization, anonymization, and combined approach (*χ*² *P* < 0.005). For the anonymized and combined approaches, this may be explained by the introduction of new, hybridized value classes to this variable by the applied generalization. Using non-uniform entropy analyses for these two cases revealed a similarity metric of 0.88 to the original data indicating a high degree of similarity on a scale from 0 to 1.0.

**Figure 2:**
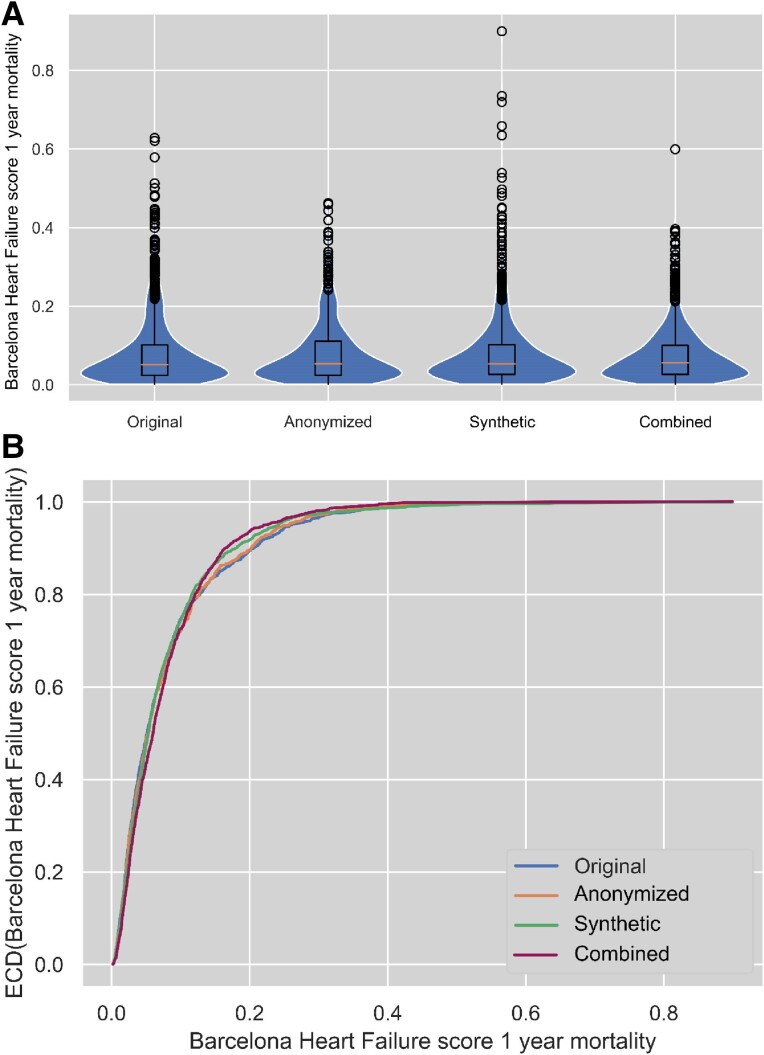
Distribution of 1-year mortality score values for Barcelona Bio Heart Failure score distributions as (*A*) violin plots and (*B*) empirical cumulative distribution function. ECD, empirical cumulative distribution.

Finally, we compared the distributions of the BioHF 1-year mortality probability for the original data set as well as for all protected data sets (see *Figure 2*). While score distributions for anonymization and synthetization do not differ significantly from the original data (cf. Supplementary material online, *Table S2*), the score distribution for the combined approach does (*P* = 0.02, *d* = 0.095).

Equivalent results were obtained for the MAGGIC score (see Supplementary material online, *Table S3*), where we found significant but very small differences for all continuous variables except body mass index in all data protection mechanisms. In contrast to the distributions of the BioHF 1-year mortality score, the MAGGIC 1-year mortality score did not show outliers in the original and protected data but showed significant, small differences (*P* = 0.005, *d* = 0.108). Similar to *Figure 2*, Supplementary material online, *Figure S3* shows the respective score distributions for the MAGGIC 1-year mortality.

### Data privacy

*Table 2* shows the results for the aforementioned privacy risk estimates, i.e. scores for linkage, singling out of individual variable values (univariate) and combination of multiple variables (multivariate) as well as inference from the Anonymeter framework, and the share metric as well as the median DCR ratio calculated with the hold-out approach.

**Table 2:** Residual risk estimates after data protection based on linkage, singling out (univariate and multivariate) and attribute inference using the Anonymeter framework and share as well as median distance-to-closest-record ratio (1: for anonymeter scores, estimated privacy risks increase with increasing deviation from 1.0; 2: for the hold-out approach, estimated privacy risks increase with increasing deviation from 0.5 for the share metric and with increasing deviation from 1.0 for the median distance-to-closest-record ratio; and 3: attack was not successful with a success rate of 0 for all attacks in all repetitions)

	BioHF			MAGGIC		
Anonymeter attacks	Synth.	Anon.	Comb.	Synth.	Anon.	Comb.
Linkage	0.00 (0.01)	**0.06 (0.03)**	0.01 (0.02)	0.01 (0.02)	**0.08 (0.01)**	0.01 (0.01)
Singling out univariate	0.00 (0.00)	0.00 (0.00)^3^	0.00 (0.00)	0.00 (0.00)	0.00 (0.00)	0.00 (0.00)
Singling out multivariate	**0.13 (0.02)**	0.02 (0.01)	0.04 (0.01)	**0.13 (0.02)**	0.01 (0.01)	0.03 (0.02)
Attribute inference	**0.02 (0.01)**	0.01 (0.01)	**0.02 (0.01)**	**0.03 (0.01)**	0.02 (0.01)	0.01 (0.01)

Hold-out Platzer *et al*.	Synth.	Anon.	Comb.	Synth.	Anon.	Comb.
Share	0.51	**0.62**	0.59	0.50	**0.71**	0.58
Median DCR ratio	0.99	**0.93**	0.95	1.00	**0.80**	0.95

The numbers in brackets show the standard deviation. The data set with the highest risk as indicated by each score or metric is highlighted in bold.

DCR, distance-to-closest-record.

As can be seen, privacy risks estimated with Anonymeter for linkage, univariate singling out, and attribute inference are generally low (between 0.0 and 0.08 of a maximum of 1.0) with anonymized data showing a slightly elevated risk of linkage (≤0.08). The estimated risk of multivariate singling out is low for the data sets protected with anonymization (≤0.02) and the combined approach (≤0.04) and elevated for the synthetization method (≤0.13). In regard to the hold-out approach, the metrics calculated for the synthetization method and the combined approach are very close to the optimum for both the share (0.50–0.51 and 0.58–0.59 with an optimum of 0.50) and the median DCR ratio (0.95–1.0 and 0.95 with an optimum of 1.0). The data protected with anonymization, however, shows elevated risk estimated with 0.62 and 0.71 for the share metric and 0.93 and 0.80 for the median DCR ratio. In summary, the synthetic data show an elevated estimated risk of multivariate singling out attacks and the anonymized data show elevated estimated risks in the hold-out approach, while the data protected with the combined method perform well in all cases. We note that the result of the combined method also implicitly fulfils the formal k-anonymity guarantee, as it was synthesized from a previously anonymized data set accordingly.

## Discussion

Privacy protection remains a significant barrier to sharing biomedical data more openly.^36^ Our work contributes to the development of innovative solutions for open data sharing by applying anonymization and synthetization techniques to a real-world clinical data set enabling real-world research as demonstrated by the calculation of two HF risk scores. Moreover, we proposed an innovative combined approach and assessed the utility for research as well as the final level of privacy protection through different means.

The main benefits of data synthetization are that it generates records that are similar but likely not identical to real-world records and that it can produce an arbitrary number of records. The main drawback of many synthetization methods is that they cannot provide formal guarantees for the degree of privacy protection achieved. The main benefit of anonymization is that it can provide such formal guarantees. However, the main drawbacks of anonymization are that it can be difficult to apply to high-dimensional data and that its outputs will be quite homogeneous. Hence, we combined both techniques sequentially to overcome these individual limitations. The combined approach also showed the lowest degree of estimated residual risks in our privacy evaluation. At the same time, the utility analysis showed a slight reduction compared with the other methods, but its result is still suitable for the development of research questions and analysis pipelines, since the major statistical properties of the original data are retained.

It is generally not recommended to provide binding thresholds for the acceptability of risks, since this ignores the context-specific aspects of a data sharing process, such as the trust of the recipient or additional controls in place. The ISO/IEC 27559:2022 standard^37^ can serve as a recent point of reference and recommends an average risk of not more than 0.1 for non-public data sets with low possibility of an attack and low to medium privacy impact, which aligns well with the setting studied in this paper. All risk scores obtained for the data set protected with the combined approach are below this threshold.

In regard to data utility, statistically significant differences with small effect sizes were found for all three protection mechanisms with the highest differences in categorical values in the combined approach. Still, effect sizes for these differences were below 0.3 indicating potentially negligible univariate deviations. The introduction of combined categories by the anonymization process would potentially require an adjusted selection of appropriate statistical tests. Since this specific generalization was chosen with the HF risk scores in mind, these changes in categories did not have any effect on the main clinical outcomes, i.e. the derived BioHF and the MAGGIC scores.

During the data pre-processing, the data set was split and tailored specifically for two different risk scores by removal of outliers and records containing missing values. Although this is not strictly necessary, it is recommended to perform quality control of the data before performing data processing like anonymization and synthetization. Outliers would likely be suppressed during anonymization anyways and they could interfere with the synthetization method, e.g. by enabling membership inference attacks. Other approaches of data pre-processing, like imputation and value caps, might also be suitable for small data sets.

Although the areas of anonymization and synthetization are very active fields of research (see Fung *et al*.^22^ and Kaabachi *et al*.^18^ for an overview), previous studies are usually characterized by the fact that they do not systematically evaluate both the utility of the protected data and their residual risks,^18^ which we have done extensively in the work presented here. Another aspect that sets our work apart is the fact that we systematically compared the results of synthetization and anonymization methods and even combined both approaches. Related work includes, for example, methodological work on the evaluation of residual risks of anonymized and synthetic data, but without considering the utility dimension.^13^ In our own previous work, we have anonymized real-world data from COVID-19 registries and studies, evaluated them extensively, and made them publicly available,^38,39^ but without considering synthetization methods.

While less complex synthetization methods generally provide adequate utility, training high parameter models such as deep neural network models on small data sets either leads to degraded utility or to overfitting, which affects data privacy negatively by simply reproducing the original records. With data sets of large numbers of features, synthesized data sets may fail to ensure utility in some or all features.^40^ Although there are no strict rules, a common ML guideline suggests having at least 10 times more examples than features.

While our study is, to the best of our knowledge, the first to systematically evaluate data anonymization, synthetization, and a combination of both approaches in a real-world context, some limitations remain. First, we used a well-defined tabular data set created for a specific use case, which means that our methods and results do not necessarily generalize to more complex scenarios. Second, due to the comparatively small input data set, the methods used for residual risk assessment may have overestimated privacy risks because of the small size of subsamples used in the evaluation process.

Regarding data set size, it is a general fact that small data sets have some limitations concerning their statistical properties: with smaller size, they tend to be less representative (sampling bias) and less suitable for training ML models (overfitting). Both anonymization and synthetization work better with larger input data sets, because these protection methods rely on extracting the distribution patterns of larger groups of individuals, thus protecting privacy. Our work on anonymization and synthetization was not affected in this regard as demonstrated by utility and statistical property analyses. Data set size also affects privacy risk assessment, where the employed frameworks typically suggest data set size orders of magnitude larger than our real-world example, as data sets are split into training and control data by some algorithms. Undersized data sets can lead to overestimation of re-identification risks. However, our thorough risk assessment showed a residual risk of not more than 0.1, which is acceptable according to ISO/IEC 27559:2022.

The aforementioned limitations underscore the need for further development of methods to estimate residual privacy risks that are applicable to synthetic and anonymized data alike.

## Conclusion

While privacy protection remains a significant challenge to the open sharing of biomedical data, our work contributes to this field by demonstrating the application of a combined anonymization and synthetization technique to a real-world clinical data set. This approach is valuable, as evidenced by the calculation of two HF risk scores, while maintaining a high level of privacy protection. The combined approach, anonymize first and synthesize second, addresses individual limitations of both techniques and presents a balanced trade-off between data utility and privacy. Generally, data utility remains suitable for research purposes, retaining major statistical properties of the original data. Our evaluation aligns with the ISO/IEC 27559:2022 standard, ensuring the privacy risks are kept within acceptable limits. Our methods, although here applied in a cardiovascular context, are generally transferable to other patient record data sets and have been developed in accordance with FAIR principles. While our findings are promising, further research is needed to generalize these methods to more complex data sets and to refine residual risk estimation techniques. This work is a stepping stone for facilitating safer and more effective data sharing in biomedical research.

## Supplementary Material

ztae083_Supplementary_Data

## Data Availability

The protected data set is available via heiDATA. heiDATA is an institutional repository for Open Research Data from Heidelberg University. Please visit: https://doi.org/10.11588/data/MXM0Q2. All relevant codes to reproduce the results may be obtained from GitHub: https://github.com/dieterich-lab/AnonymizeAndSynthesize.
