# Supplementary material for: Anonymize or synthesize? Privacy-preserving methods for heart failure score analytics
Source: Eur Heart J Digit Health. 2024 Nov 20;6(1):147–54. doi: 10.1093/ehjdh/ztae083 (PMC11750188; doi:10.1093/ehjdh/ztae083)
Supplement: ztae083_Supplementary_Data [file ztae083_supplementary_data.pdf]

Supplementary Information

Anonymize or Synthesize? - Privacy-Preserving Methods for Heart Failure Score Analytics

Tim I. Johann Karen Otte Fabian Prasser
Christoph Dieterich

June 28, 2024

S1 Evaluation of PCA in Synthetic Data

We investigated the cross-correlation structure in the original and synthetic data sets by performing a principal component analysis (PCA) on the respective input variables as listed in Table S1. All categorical variables were one-hot encoded. All continuous variables were centered and scaled. PCA was performed on the original data only and the synthetic data were projected on PC1 and 2 (see Figures S1 and S2). Our data synthesis strategy yields a very similar 2D-density distribution as presented in both plots.

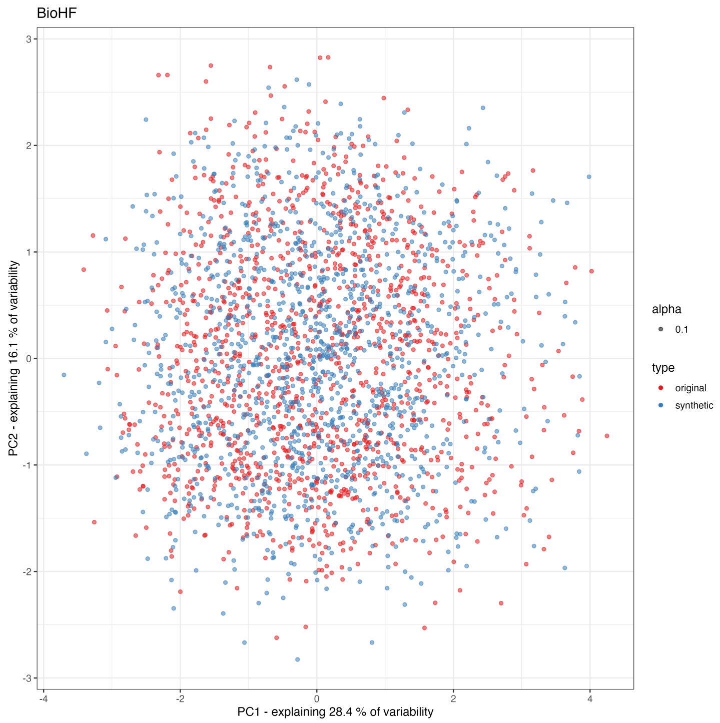

Figure S1: **PCA Analysis of synthetic BioHF scores.** A comparison of the original BioHF input variables with the synthetic data. PC1 represents age (-0.57), estimated GFR (+0.59) and haemoglobin (+0.51). PC2 represents left ventricular ejection fraction (-0.9), estimated GFR (-0.24) and sodium (-0.16)

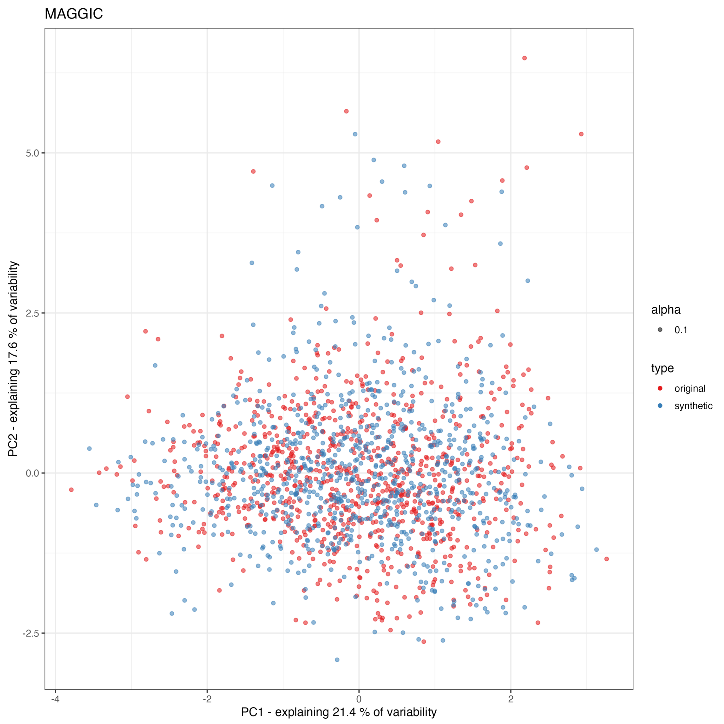

Figure S2: **PCA Analysis of synthetic MAGGIC scores.** A comparison of the original MAGGIC input variables with the synthetic data. PC1 represents age (-0.44), systolic blood pressure (-0.65) and left ventricular ejection fraction (-0.6). PC2 represents creatinine (+0.7), bmi (-0.26) and age (+0.56)

OpenEHR Template	Data Items *
Personal Data	Age (B, M)
Patient history	Sex (B, M)
	BMI (M)
	Systolic blood pressure (M)
	NYHA dysfunctional class (B, M)
	Current smoking (M)
	Time of first diagnosis of HF (M)
	Diabetes mellitus (M)
	Chronic lung disease (M)
Medication	Betablocker (B, M)
	ACEi/ARB (B, M)
	Statin (B)
	Loop diuretic (B)
Echocardiography	LV ejection fraction (B, M)
Laboratory Data	Creatinine (M) [LOINC 14682-9, 2160-0]
	Sodium (B) [LOINC 2951-2]
	Hemoglobin (B) [LOINC 718-7, 30350-3]
	Estimated GFR (B) [LOINC 62238-1]

Table S1: Record entries of the UCC dataset (cf. [5]). The letters in parentheses denote which score they contribute to. B stands for BioHF and M for MAGGIC. The original height and weight entries have been replaced by 'BMI', while 'Year of birth', reported in [5] had been tranlated to 'Age' in the basic dataset.

S2 Utility Statistics

Table S2 and S3 detail the comparative statistics between original and synthetic, anonymized and combined datasets respectively. For continuous variables the two-sample Kolmogorov-Smirnov-Test was applied, i.e. each continuous variable distribution of a processed dataset was tested against the distribution of the respective variable in the original dataset. The same procedure was done with categorical and boolean variables using the χ^2-test.

Statistics were calculated in Python 3.10 using the *scipy.stats* package and utilities in *numpy* and *pandas*.

S2.1 Statistical Interpretation

The two-sample Kolmogorov-Smirnov method was used to quantify similarity of original and processed distributions of variable and result values. In this test two distributions can be said to be derived from the same population (the null-hypothesis, H_0), if the KS statistic d is *below* a critical value $d(\alpha)$, where α is the significance level chosen by the statistician, in this study we used the typical 5%, meaning that we want to have a likelyhood of 95% for the observed deviations to be attributed to the sampling process alone. The critical value is calculated as $d_{crit}(\alpha, n, m) = c(\alpha) \cdot \sqrt{\frac{n+m}{n \cdot m}}$ where $c(\alpha)$ is the value of the inverse Kolmogorov distribution at the chosen significance level α and n and m are the sample sizes of the data [2].

With the sample sizes having an at most marginal difference we can assume $n = m = N$ and the formula for the critical value simplifies to $d_{crit} = c(\alpha) \cdot \sqrt{\frac{2}{N}}$. The value of $c(\alpha)$ for $\alpha = 0.05$ is 1.358. This yields the critical values for a sample size of 1326 (BioHF) of $d_{crit}(\alpha = 5\%, N = 1326) = 0.05274$ and for 890 samples $d_{crit}(\alpha = 5\%, N = 890) = 0.064375$.

The effect size magnitude measure **Cohen's d** quantifies the effect of a possibly existing alternative hypothesis to alter the mean value of one sample to the other. To dismiss the alternative hypothesis, small (< 0.2) or very small (< 0.01) d-values are necessary ([1], [4]).

For categorical variable frequency distributions, the χ^2 p-value can be compared to the chosen significance level. The p-value denotes the probability for the observed differences to be due to random sampling from the same distribution [2]. As for the two-sample KS test, the null-hypothesis is that the two distributions originate from the same distribution, and when encountering a p-value larger than a chosen significance level we cannot reject H_0 in favour of the alternative.

Cramér's V is a normalised quantifier for the *association* of two categorical variables (or, in this case, samples). The value of Cramér's V varies from 0 for no association to 1 of complete association [3].

						Original vs. Synthetic			Original vs. Anonymized			Original vs. Combined		
		original, N=1326	synthetic, N=1326	anonymized, N=1324	combined, N=1324	KS p-value	KS d value	Cohens D	KS p-value	KS d value	Cohens D	KS p-value	KS d value	Cohens D
age		67.47 (14.28)	67.63 (14.04)	67.03 (13.42)	66.76 (13.70)	0.91	0.02	-0.01	0.009	0.064	0.032	0.032	0.055	0.051
lvef_m		41.83 (14.89)	41.45 (15.24)	41.16 (13.59)	41.11 (14.16)	0.52	0.03	0.02	0.01	0.063	0.047	0.018	0.059	0.049
sodium_m		139.17 (3.25)	139.14 (3.23)	139.16 (2.70)	139.11 (2.71)	0.00	0.09	0.010	0.000	0.086	0.000	0.000	0.113	0.017
hb_m		13.07 (2.10)	12.98 (2.10)	13.02 (1.79)	13.00 (1.79)	0.01	0.06	0.04	0.000	0.082	0.027	0.01	0.063	0.037
egfr_m		59.76 (24.32)	59.30 (24.37)	58.90 (22.81)	58.35 (22.48)	0.78	0.03	0.02	0.103	0.047	0.037	0.074	0.05	0.06
biohf_v1_1		0.08 (0.09)	0.08 (0.09)	0.08 (0.08)	0.07 (0.07)	0.16	0.04	0.03	0.737	0.027	0.01	0.02	0.06	0.095
		orig. Freq. (ratio)	synt. Freq. (ratio)	anon. Freq. (ratio)	comb. Freq. (ratio)	χ^2 p-value	χ^2	Cramér's V	χ^2 p-value	χ^2	Cramér's V	χ^2 p-value	χ^2	Cramér's V
gender	m	948 (0.71)	955 (0.72)	946 (0.71)	920 (0.69)	0.668	0.183	0.012	0.972	0.001	0.001	0.113	2.519	0.044
	f	378 (0.29)	371 (0.28)	378 (0.29)	404 (0.31)									
nyha	I	247 (0.19)	263 (0.20)	142 (0.11)	144 (0.11)	0.039	8.375	0.046	0	86.413	0.147	0	201.89	0.225
	II	558 (0.42)	567 (0.43)	454 (0.34)	427 (0.32)									
	III	479 (0.36)	468 (0.35)	446 (0.34)	470 (0.35)									
	IV	42 (0.03)	28 (0.02)	12 (0.01)	6 (0.00)									
	{I, II}			208 (0.16)	213 (0.16)									
	{III, IV}			62 (0.05)	64 (0.05)									
beta	0	272 (0.21)	264 (0.20)	250 (0.20)	223 (0.17)	0.582	0.303	0.015	0.367	0.813	0.025	0.003	8.723	0.083
	1	1054 (0.79)	1062 (0.80)	1030 (0.80)	1056 (0.83)									
furosemide1	0	452 (0.34)	452 (0.34)	426 (0.33)	438 (0.34)	1	0	0	0.588	0.294	0.015	0.969	0.002	0.001
	1	874 (0.66)	874 (0.66)	850 (0.67)	845 (0.66)									
statin	0	508 (0.38)	498 (0.38)	484 (0.38)	483 (0.37)	0.571	0.322	0.016	0.708	0.14	0.01	0.528	0.399	0.018
	1	818 (0.62)	828 (0.62)	796 (0.62)	806 (0.63)									
acei_arb	0	406 (0.31)	404 (0.30)	372 (0.30)	392 (0.31)	0.905	0.014	0.003	0.403	0.699	0.024	0.92	0.01	0.003
	1	920 (0.69)	922 (0.70)	886 (0.70)	883 (0.69)									

Table S2: Statistical fidelity of distributions of variables used to calculate BioHF.

		original, N=890	synthetic, N=890	anonymized, N=888	combined, N=888	Original vs. Synthetic			Original vs. Anonymized			Original vs. Combined		
						KS p-value	KS d value	Cohens D	KS p-value	KS d value	Cohens D	KS p-value	KS d value	Cohens D
age		65.52 (14.67)	64.82 (15.60)	64.93 (13.14)	65.84 (13.44)	0.392	0.043	0.046	0.001	0.089	0.043	0.301	0.046	-0.022
bmi		28.45 (6.05)	28.16 (5.84)	28.29 (5.18)	28.46 (5.00)	0.15	0.054	0.049	0.115	0.056	0.028	0.105	0.057	-0.002
sys_bp_m		125.06 (22.47)	123.15 (22.92)	124.26 (16.88)	125.26 (16.62)	0.005	0.082	0.084	0.001	0.094	0.040	0.000	0.096	-0.011
lvef_m		41.59 (14.76)	40.65 (15.25)	40.77 (13.11)	40.41 (13.38)	0.25	0.048	0.063	0.043	0.065	0.059	0.015	0.074	0.084
creatinine_m		122.86 (85.74)	122.19 (85.45)	115.55 (44.57)	116.35 (45.19)	0.027	0.07	0.008	0.003	0.085	0.107	0.003	0.085	0.095
maggic_score_1		0.14 (0.11)	0.13 (0.09)	0.13 (0.10)	0.13 (0.08)	0.067	0.062	0.089	0.633	0.037	0.068	0.005	0.085	0.108
		orig. Freq. (ratio)	synt. Freq. (ratio)	anon. Freq. (ratio)	comb. Freq. (ratio)	χ^2 p-value	χ^2	Cramér's V	χ^2 p-value	χ^2	Cramér's V	χ^2 p-value	χ^2	Cramér's V
gender	m	639 (0.72)	621 (0.70)	638 (0.72)	635 (0.72)	0.189	1.726	0.044	0.974	0.001	0.001	0.849	0.036	0.006
	f	251 (0.28)	269 (0.30)	250 (0.28)	253 (0.28)									
nyha	I	211 (0.24)	219 (0.25)	116 (0.13)		0.46	2.585	0.031	0	253.835	0.309			
	II	377 (0.42)	391 (0.44)	264 (0.30)										
	III	272 (0.31)	251 (0.28)	206 (0.23)										
	IV	30 (0.03)	29 (0.03)	2 (0.00)										
	{I, II}			206 (0.23)										
	{III, IV}			94 (0.11)										
smoking	0	773 (0.87)	770 (0.87)	760 (0.88)	773 (0.89)	0.768	0.087	0.01	0.309	1.034	0.035	0.113	2.51	0.054
	1	117 (0.13)	120 (0.13)	104 (0.12)	100 (0.11)									
diabetes	0	625 (0.70)	654 (0.73)	604 (0.71)	617 (0.71)	0.028	4.849	0.074	0.583	0.301	0.019	0.5	0.454	0.023
	1	265 (0.30)	236 (0.27)	246 (0.29)	249 (0.29)									
copd	0	790 (0.89)	801 (0.90)	778 (0.90)	798 (0.92)	0.219	1.511	0.041	0.201	1.633	0.043	0.001	12.074	0.118
	1	100 (0.11)	89 (0.10)	86 (0.10)	70 (0.08)									
hf_gt_18_months	0	176 (0.20)	163 (0.18)	160 (0.19)	150 (0.17)	0.26	1.269	0.038	0.388	0.745	0.029	0.059	3.552	0.064
	1	714 (0.80)	727 (0.82)	698 (0.81)	713 (0.83)									
beta	0	200 (0.22)	160 (0.18)	178 (0.21)	183 (0.22)	0	12.192	0.117	0.295	1.098	0.036	0.482	0.494	0.024
	1	690 (0.78)	730 (0.82)	668 (0.79)	668 (0.78)									
acei_arb	0	258 (0.29)	261 (0.29)	240 (0.28)	273 (0.32)	0.825	0.049	0.007	0.587	0.296	0.019	0.057	3.63	0.065
	1	632 (0.71)	629 (0.71)	612 (0.72)	581 (0.68)									

Table S3: Statistical fidelity of distributions of variables used to calculate the MAGGIC score.

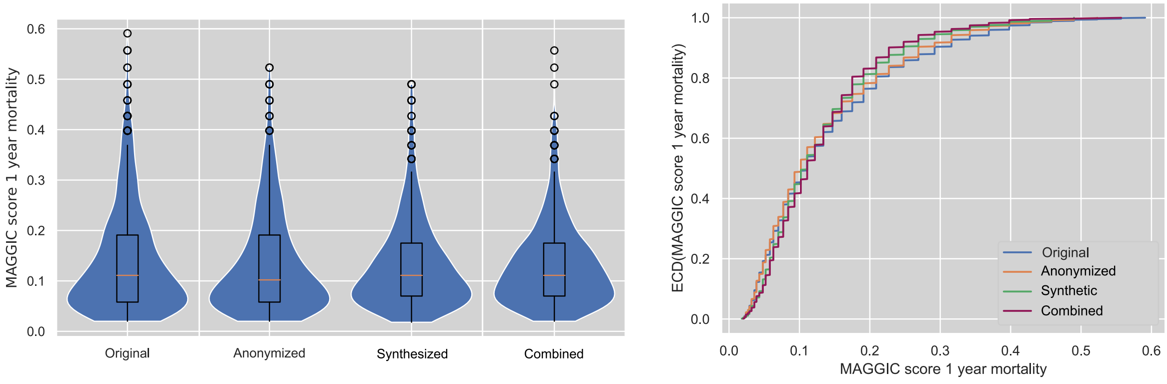

(a) MAGGIC scores: Violin plot

(b) MAGGIC scores: ECD plot

Figure S3: Distribution of 1 year mortality score values for MAGGIC score distributions. (a) Four different score distributions are presented as violin plots based on the original data set, the anonymized, the synthesized and the combined approach (synthesis followed by anonymization). (b) Empirical Cumulative Distribution (ECD) function of the same data as in (a).

References

[1] Jacob Cohen. A power primer. *Psychological Bulletin*, 112(1):155–159, 1992.

[2] William Jay Conover. *Practical nonparametric statistics*. Wiley series in probability and statistics. Wiley, New York, NY, 3. ed edition, 1999.

[3] Harald Cramér. *Mathematical Methods of Statistics (PMS-9)*. Princeton University Press, December 1946.

[4] Shlomo S. Sawilowsky. New effect size rules of thumb. *Journal of Modern Applied Statistical Methods*, 8(2):597–599, November 2009.

[5] Kim K Sommer, Ali Amr, Udo Bavendiek, Felix Beierle, Peter Brunecker, Henning Dathe, Jürgen Eils, Maximilian Ertl, Georg Fette, Matthias Gietzelt, Bettina Heidecker, Kristian Hellenkamp, Peter Heuschmann, Jennifer D E Hoos, Tibor Kesztyüs, Fabian Kerwagen, Aljoscha Kindermann, Dagmar Krefting, Ulf Landmesser, Michael Marschollek, Benjamin Meder, Angela Merzweiler, Fabian Prasser, Rüdiger Pryss, Jendrik Richter, Philipp Schneider, Stefan Störk, and Christoph Dieterich. Structured, harmonized, and interoperable integration of clinical routine data to compute heart failure risk scores. *Life (Basel)*, 12(5):749, May 2022.
